# A Prospective Evaluation of Postoperative Readmissions After Outpatient Hand and Upper Extremity Surgery

**DOI:** 10.7759/cureus.15247

**Published:** 2021-05-26

**Authors:** Kristin Sandrowski, Moody Kwok, Greg Gallant, Jack Abboudi, Robert Takei, Samir Sodha, Daren Aita, Mark Wang, Christopher Jones, Pedro K Beredjiklian

**Affiliations:** 1 Division of Hand Surgery, Rothman Orthopaedic Institute, Philadelphia, USA; 2 Division of Hand Surgery, Rothman Orthopaedic Institute, Paramus, USA; 3 Orthopaedic Surgery, Hackensack University Medical Center, New York, USA

**Keywords:** readmission, outpatient, surgery, hand, ambulatory surgery

## Abstract

Introduction

Hand and upper extremity surgeries are largely performed in free-standing ambulatory surgery centers (ASCs). Rates of unexpected hospitalizations or visits to the urgent care or emergency departments in the month following hand and upper extremity surgery have been widely varied in the literature. We prospectively followed patients after hand and upper extremity outpatient surgery to determine the rate of unplanned health care utilization with the hypothesis that hospital admissions, emergency room visits, and urgent care center visits would be higher than the rates currently reported by retrospective studies.

Methods

All patients undergoing outpatient hand and upper extremity surgery by five hand surgeons were prospectively followed to monitor for hospital readmissions, emergency room visits, and urgent care presentations. The patients’ postoperative course was evaluated for direct transfers from the surgical center to the hospital. In addition, any urgent care or emergency room visits and hospital admissions for the first month after surgery were tabulated. Points of review of the patients’ postoperative course included the following: (1) phone contact on the first postoperative day, (2) routine ASC postoperative phone calls two to three days postoperatively, (3) first postoperative office at approximately one to two weeks, and (4) phone contact or office evaluation one-month postoperatively based on surgeon preference for follow-up.

Results

A total of 583 patients were identified for participation, of whom 22 patients were excluded; thus, 561 patients were included for evaluation, with 47.2% women (n=265) and 52.8% men (n=296). The average age was 54 years (range: 14-102 years). Nine (1.6%) patients presented postoperatively for further evaluation at an urgent care or hospital (95% C.I. 0.8-3.1%). Five patients presented to an emergency room and four patients presented to an urgent care facility. Of those patients, two were admitted to the hospital due to shortness of breath (0.35%; 95% CI: -0.08 to 1.4%). Emergency room and urgent care visits that did not lead to admission accounted for 1.25% (95% CI: 0.6-2.6%). No patients were transferred from the ASC to the hospital or emergency room.

Conclusion

There was a low rate of postoperative utilization of urgent care and emergency room services with hand and upper extremity surgery performed at free-standing, ASCs. Hospital readmissions were rare, and no patients required transfer from an ambulatory care center to the hospital. Outpatient hand and upper extremity surgery is safe in an ambulatory care center, with low postoperative transfers and readmissions in the month following surgery.

## Introduction

The number of surgical procedures performed at free-standing outpatient ambulatory surgery centers (ASCs) continues to increase as a result of improved cost-effectiveness, patient convenience, and overall efficiency [1]. With this increasing demand for ASC utilization, patient safety is paramount and highlights the need for appropriate patient and procedure criteria to reduce unplanned utilization of acute care resources in the postoperative period. Although hospital transfer or readmission at the time of ASC discharge appears to be a rare event, eliminating all unplanned acute care transfers continues to be an important risk management goal from a health safety, as well as cost expenditure standpoint. In response to an overall goal of eliminating any unplanned hospital readmission, Medicare, in accordance with the Affordable Care Act, has instituted financial penalties for hospitals with 30-day readmission rates that exceed a predetermined value [2]. Accordingly, postoperative readmissions following outpatient surgery have come under increased scrutiny. A better understanding of the rate and reasons for acute care utilization after ambulatory surgery will enable further refinement of patient selection criteria [3].

Current studies reporting the rate of adverse events following outpatient orthopedic procedures performed at free-standing ASCs are sparse and wide-ranging (0.05-20%) [1]. This is in part due to the retrospective nature of existing studies - often relying on office notes for reporting of readmissions - thus subject to recall bias, incomplete records, and inability to accurately account for emergency room and urgent care encounters outside of a specific hospital system. Moreover, the reason for such encounters is often unknown or extracted from coding databases in these retrospective studies, limiting interpretation of the readmission data. Patient factors including age, obesity, significant medical comorbidities, and ASA (American Society of Anesthesiologists) grade, have been identified as significant risk factors for readmission. Procedural factors such as the type of anesthesia, length of procedure, operator, and facility surgical volume have also been associated with a risk of readmission [4-9].

To date, an accurate assessment of the rate and reasons for postoperative acute care utilization following outpatient surgery in ASCs is lacking. The aim of this study is to prospectively follow patients after hand surgery performed at an outpatient ASC to determine (1) the rate of acute care utilization in the immediate postoperative period 30 days after surgery, (2) the reasons for acute care visits, and (3) the associated patient and procedural risk factors. We hypothesized that hospital admissions, emergency room visits, and urgent care center visits would be higher than the rates currently reported by retrospective studies.

## Materials and methods

All patients undergoing outpatient upper extremity surgery by five fellowship-trained hand surgeons at one free-standing ASC were prospectively followed between April and August of 2017. Patients were excluded if they did not have at least one-month follow-up or if they wished to be excluded from the study. The first 30 days course after surgery for these patients were monitored for transfers of care from the ASC, hospital admissions, emergency room visits, and urgent care presentations.

Any same-day transfers from the ASC to the hospital were recorded. Scheduled points of postoperative patient contact included (1) phone contact on the first postoperative day, (2) routine ASC postoperative phone calls two to three days postoperatively, (3) first postoperative office at approximately one to two weeks, and (4) phone contact or office evaluation one-month postoperatively based on surgeon preference for follow-up. In addition, phone calls and other communications with the patients, as well as office and ASC electronic medical records were monitored. At each interaction, patients were asked whether they had visited an emergency room or urgent care or had been admitted to a hospital. The reasons and timing for any ASC transfer, hospital admission, emergency visit, and urgent care visit were recorded.

## Results

A total of 583 patients were identified for participation. Eight patients asked to be excluded from the study and 14 patients were excluded for lack of one-month follow-up. The remaining 561 patients (96.2% of the original group) were included for evaluation, with 47.2% women (n=265) and 52.8% men (n=296). The average age was 54 years (range: 14-102 years) and BMI was 28.7 (range: 14.73-48.65). Surgeries were performed from elbow to fingers, with 30.8% of the procedures involving bone work and 69.2% involving soft tissue only. A total of 41 patients underwent more than one surgical procedure during the index operation (e.g., carpal tunnel and trigger finger releases) for a total of 602 procedures (Table 1).

**Table 1:** Surgical procedures ORIF, open reduction and internal fixation; CMC, carpometacarpal; SL/LT, scapholunate/lunotriquetral ligaments; TFCC, triangular fibrocartilage complex; DIP, distal interphalangeal; I&D, irrigation and debridement; ECU, extensor carpi ulnaris; ROH, removal of hardware; DRUJ, distal radioulnar joint; PIP, proximal interphalangeal

Procedure	Number of Cases	Procedure	Number of Cases
Carpal tunnel release	134	Ulnar nerve transposition	3
Trigger finger release	95	Foreign body removal	3
Mass excision	49	Rotator cuff repair	3
Distal radius ORIF	47	Mallet finger repair	2
Metacarpal ORIF	39	Distal biceps repair	2
Cubital tunnel release	26	Humerus ORIF	2
CMC collateral ligament reconstruction	21	SL/LT ligament reconstruction	2
CMC arthroplasty	20	Nail excision	2
Phalanx ORIF	18	Proximal row carpectomy	2
DeQuervain’s release	17	Scaphoidectomy	2
TFCC repair	15	Ray resection	1
Epicondyle debridement	13	Wrist fusion	1
Dupuytren’s release	11	Sagittal band reconstruction	1
Tendon repair	10	Triceps repair	1
Carpal bone ORIF	9	DIP fusion	1
I&D	9	ECU sheath reconstruction	1
Elbow ORIF	7	Elbow contracture release	1
Elbow/wrist ROH	6	Skin graft	1
Finger amputation	5	Nerve repair	1
Tenolysis	4	Volar plate repair	1
Olecranon bursa excision	4	Olecranon osteophyte excision	1
Ulna hemiresection	4	DRUJ ORIF	1
Wrist arthroscopy	3	PIP fusion	1

Nine (1.6%) patients presented within one month from surgery for evaluation at an urgent care or hospital (95% CI: 0.8-3.1%) (Table 2). Five patients presented to an emergency room and four to an urgent care facility. Of those patients, two (0.35%) were admitted to the hospital (95% CI: -0.08 to 1.4%) and both admissions were related to shortness of breath, with one of them due to pneumonia. The remaining emergency room and urgent care visits that did not lead to admission accounted for 1.2% (95% CI: 0.6-2.6%). Six patients returned due to complications at their surgical site and three due to anesthesia-related complications (patients 3, 5, and 6 underwent local blocks with intravenous sedation). Two of the nine patients received regional blocks, and the rest received local blocks with intravenous sedation.

**Table 2:** Patients who presented for urgent care and emergency room visits BMI, body mass index; CAD, coronary artery disease; CTR, carpal tunnel release; HTN, hypertension; ORIF, open reduction and internal fixation; POD, postoperative day; UCL, ulnar collateral ligament

Patient	Age (years)	Gender	BMI	Comorbidities	Smoker	Surgical Procedure	Hospital or Urgent Care Visit	Readmission	Reason for Visit and POD
1	49	F	31.9	Anxiety	No	CTR, DeQuervain’s release	Emergency room	No	Surgical site infection, POD 17
2	73	M	25.1	Prostate cancer	No	Finger mass excision	Urgent care	No	Swelling and pain, POD 2
3	86	M	29.4	Abdominal aortic aneurysm, diabetes, HTN	No	Trigger finger releases	Emergency room	Yes	Shortness of breath, POD 9
4	43	F	25.7	Asthma, migraines, Arnold Chiari malformation	No	Phalanx ORIF	Urgent care	No	Surgical pin migration, POD 22
5	76	M	28.5	Chronic bronchitis, CAD, HTN	Former	Fasciectomy	Emergency room	Yes	Shortness of breath, pneumonia, POD 1
6	28	M	27.3	Asthma	Former	Thumb UCL reconstruction	Emergency room	No	Vertigo, POD 3
7	55	M	34.3	Sleep apnea, HTN, hyperlipidemia	No	Wrist mass excision	Urgent care	No	Surgical site infection, POD 12
8	41	M	24.4	Lyme disease	No	Wrist mass excision	Emergency room	No	Fall and wound dehiscence, POD 12
9	55	M	34.7	Traumatic brain injury, diabetes	no	Distal radius ORIF	Urgent care	No	Patient removal of cast, POD 8

## Discussion

Hand and upper extremity surgery has shifted to the ambulatory setting due to cost savings, efficiency, and convenience for patients. Postoperative utilization of urgent care centers and hospitals negatively affects the patient experience, poses a safety risk, increases the episode of care cost, and has the potential to limit reimbursement [1,10-20]. Our goal was to study the utilization of acute care services prospectively to attempt to more accurately assess postoperative acute care visits and their reasons after hand and upper extremity surgery. These data can help improve ASC surgical selection criteria and identify areas for process improvement.

Understanding reasons for postoperative acute care utilization can be important for decreasing future readmissions by targeting preoperative patient education. Increased comorbidities, BMI, obesity, surgical time, and ASA physical status classification are associated with an increased risk for readmission, suggesting the importance of patient selection for ambulatory surgery [5,7,20,21]. Pak et al. found that outpatient utilization of spine clinics was the only factor independently associated with a reduced likelihood of emergency room utilization suggesting, the importance of appropriate follow-up and patient counseling about office resources available for postoperative concerns [14]. In addition to streamlining the patient experience by establishing appropriate postoperative resources, the ability to decrease hospital and urgent care visits would lead to decreased health care costs. Coley et al. evaluated mean charges for patients with unanticipated admissions, with an average cost of $1,869 ± $4,553 per pain-related visit and $12,000 +/- $36,886 for non-pain related readmission [5].

We identified five studies specifically looking at readmission following outpatient hand surgery. All were retrospective studies and two of these were database studies. There was a wide variability between studies, with reported postoperative utilization as low as 0.07% to as high as 9% [1-4,9]. Curtin and Hernandez-Boussard reported a 9% readmission rate, but only monitored patients who underwent distal radius fracture treatment, which is not representative of all hand and upper extremity surgery [11]. Similarly, Menendez and Ring retrospectively reviewed patients who only underwent carpal tunnel and trigger finger surgery and found a 3% emergency room utilization [7]. Goyal et al. reported a low postoperative emergency room utilization of 0.07% [12]. The reasons for this low number may be explained by the fact that these patients were only followed for seven days after surgery and the retrospective nature of the study. In two database studies, Donato et al. and Noureldin et al. reported readmission rates of 0.88% and 1.2%, respectively [6,13]. While a large number of patients were included, these studies were retrospective and relied on admissions recorded in their database.

Of the above cited studies, the reported rates of acute care utilization in outpatient hand surgery varied, and the reasons for seeking care were similar, with pain and wound complication being most frequently reported [1-4]. Unfortunately, as the majority of studies have been retrospective, the cause of presentation and readmission was often unknown, thus limiting interpretation of the data. Noureldin et al. reported postoperative infection and pain as the most common reason for presentation, but over a third of readmissions had no reason identified [13]. Similarly, Curtin and Hernandez-Boussard reported distal radius as one of the most common emergency room codes for readmission, not providing any insight into the actual reason for admission [11]. By monitoring patients prospectively, we were able to identify the cause of presentation and reason for readmission. In our experience, we found that six of the nine patients who presented to an urgent care or emergency room had a surgical site issue and only one of the nine had uncontrolled pain. None of these patients required readmission.

Our study prospectively followed a representative population of patients undergoing a variety of hand surgery procedures for 30 days and accounted for all postoperative presentations for urgent care and emergency room services and found a 1.6% rate of utilization, a 0.35% rate of hospital admission, and no ASC transfers. While some hospital admissions are unavoidable, some may be preventable. In addition to appropriate patient selection, patient education and improved availability of office resources might decrease rates of unintended acute care utilization.

Limitations of this study include potential recall bias of the patient as we relied on the patients to report hospital or urgent care visits. We attempted to control for this by contacting patients at four time points postoperatively and by monitoring all communication with the office and surgical center in the 30 days postoperatively. Additionally, our study had a limited number of patients when compared to larger retrospective studies. In addition, there is a selection bias as all of these patients were indicated for surgery in an ASC. Obviously, sicker patients and those not felt to be appropriate for surgery in an ASC were excluded from the study. Finally, we were underpowered when it came to evaluating for incidences of hospital transfer from an ambulatory care center.

## Conclusions

By following patients prospectively, we were better able to determine the rate and reason of both urgent care and emergency room visits. We conclude that outpatient hand and upper extremity surgery is safe in an ambulatory care center, with low ASC transfer rate, postoperative utilization of acute care services, and hospital admissions in the month following surgery.

## References

[1] Goldfarb CA, Bansal A, Brophy RH (2017). Ambulatory surgical centers: a review of complications and adverse events. J Am Acad Orthop Surg.

[2] Postel M, Frank PN, Barry T, Satou N, Shemin R, Benharash P (2014). The cost of preventing readmissions: why surgeons should lead the effort. Am Surg.

[3] Martín-Ferrero MÁ, Faour-Martín O, Simon-Perez C, Pérez-Herrero M, de Pedro-Moro JA (2014). Ambulatory surgery in orthopedics: experience of over 10,000 patients. J Orthop Sci.

[4] Bhattacharyya N, Kepnes LJ (2014). Revisits and postoperative hemorrhage after adult tonsillectomy. Laryngoscope.

[5] Coley KC, Williams BA, DaPos SV, Chen C, Smith RB (2002). Retrospective evaluation of unanticipated admissions and readmissions after same day surgery and associated costs. J Clin Anesth.

[6] Donato DP, Kwok AC, Bishop MO, Presson AP, Agarwal JP (2017). Unplanned readmission in outpatient hand surgery: an analysis of 23,613 patients in the NSQIP data set. Eplasty.

[7] Menendez ME, Ring D (2016). Emergency department visits after hand surgery are common and usually related to pain or wound issues. Clin Orthop Relat Res.

[8] Sherman SL, Lyman S, Koulouvaris P, Willis A, Marx RG (2008). Risk factors for readmission and revision surgery following rotator cuff repair. Clin Orthop Relat Res.

[9] Voskuijl T, Hageman M, Ring D (2014). Higher Charlson Comorbidity Index Scores are associated with readmission after orthopaedic surgery. Clin Orthop Relat Res.

[10] Bykowski MR, Sivak WN, Cray J, Buterbaugh G, Imbriglia JE, Lee WP (2011). Assessing the impact of antibiotic prophylaxis in outpatient elective hand surgery: a single-center, retrospective review of 8,850 cases. J Hand Surg Am.

[11] Curtin CM, Hernandez-Boussard T (2014). Readmissions after treatment of distal radius fractures. J Hand Surg Am.

[12] Goyal KS, Jain S, Buterbaugh GA, Imbriglia JE (2016). The safety of hand and upper-extremity surgical procedures at a freestanding ambulatory surgery center: a review of 28,737 cases. J Bone Joint Surg Am.

[13] Noureldin M, Habermann EB, Ubl DS, Kakar S (2017). Unplanned readmissions following outpatient hand and elbow surgery. J Bone Joint Surg Am.

[14] Pak LM, Fogel HA, Chaudhary MA (2018). Outpatient spine clinic utilization is associated with reduced emergency department visits following spine surgery. Spine (Phila Pa 1976).

[15] Cagle PJ Jr, Reams M, Agel J, Bohn D (2014). An outcomes protocol for carpal tunnel release: a comparison of outcomes in patients with and without medical comorbidities. J Hand Surg Am.

[16] Fortier J, Chung F, Su J (1998). Unanticipated admission after ambulatory surgery--a prospective study. Can J Anaesth.

[17] Fox JP, Vashi AA, Ross JS, Gross CP (2014). Hospital-based, acute care after ambulatory surgery center discharge. Surgery.

[18] Harness NG, Inacio MC, Pfeil FF, Paxton LW (2010). Rate of infection after carpal tunnel release surgery and effect of antibiotic prophylaxis. J Hand Surg Am.

[19] Hu K, Zhang T, Hutter M, Xu W, Williams Z (2016). Thirty-day perioperative adverse outcomes after peripheral nerve surgery: an analysis of 2351 patients in the American College of Surgeons National Surgical Quality Improvement Program Database. World Neurosurg.

[20] Mezei G, Chung F (1999). Return hospital visits and hospital readmissions after ambulatory surgery. Ann Surg.

[21] Vorhies JS, Wang Y, Herndon JH, Maloney WJ, Huddleston JI (2012). Decreased length of stay after TKA is not associated with increased readmission rates in a national Medicare sample. Clin Orthop Relat Res.

